# Quality Assessment of Some Essential Children's Medicines Sold in Licensed Outlets in Ashanti Region, Ghana

**DOI:** 10.1155/2018/1494957

**Published:** 2018-05-21

**Authors:** Grace Frimpong, Kwabena Ofori-Kwakye, Noble Kuntworbe, Kwame Ohene Buabeng, Yaa Asantewaa Osei, Mariam El Boakye-Gyasi, Ofosua Adi-Dako

**Affiliations:** ^1^Department of Pharmaceutics, Faculty of Pharmacy and Pharmaceutical Sciences, College of Health Sciences, Kwame Nkrumah University of Science and Technology, Kumasi, Ghana; ^2^Department of Pharmaceutical Sciences, Faculty of Medicine and Health Sciences, Kumasi Technical University, Kumasi, Ghana; ^3^Department of Pharmacy Practice, Faculty of Pharmacy and Pharmaceutical Sciences, College of Health Sciences, Kwame Nkrumah University of Science and Technology, Kumasi, Ghana; ^4^Department of Pharmaceutics and Microbiology, University of Ghana School of Pharmacy, Legon, Accra, Ghana

## Abstract

The quality of 68 samples of 15 different essential children's medicines sold in licensed medicine outlets in the Ashanti Region, Ghana, was evaluated. Thirty-two (47.1%) of the medicines were imported, mainly from India (65.6%) and the United Kingdom (28.1%), while 36 (52.9%) were locally manufactured. The quality of the medicines was assessed using content of active pharmaceutical ingredient (API), pH, and microbial limit tests, and the results were compared with pharmacopoeial standards. Twenty-six (38.2%) of the samples studied passed the official content of API test while 42 (61.8%) failed. Forty-nine (72.1%) of the samples were compliant with official specifications for pH while 19 (27.9%) were noncompliant. Sixty-six (97.1%) samples passed the microbial load and content test while 2 (2.9%) failed. Eighteen (26.5%) samples passed all the three quality evaluation tests, while one (1.5%) sample (CFX1) failed all the tests. All the amoxicillin suspensions tested passed the three evaluation tests. All the ciprofloxacin, cotrimoxazole, flucloxacillin, artemether-lumefantrine, multivitamin, and folic acid samples failed the content of API test and are substandard. The overall API failure rate for imported products (59.4%) was comparable to locally manufactured (63.9%) samples. The results highlight the poor quality of the children's medicines studied and underscore the need for regular pharmacovigilance and surveillance systems to fight this menace.

## 1. Introduction

Children's medicines are generally intended for the diagnostic use, management, and treatment of diseases of children up to 12 years of age. Access to affordable and quality children's medicines in developing countries such as Ghana is important in reducing the high morbidity and mortality associated with malaria and other infectious diseases of children. To address the special medication needs of children up to age 12, the World Health Organization (WHO) in October 2007 introduced the WHO Model List of Essential Medicines for children (EMLc), with the current edition published in March 2017 (6th Edition). The EMLc consists of a core list and a supplementary list of medicines. The core list contains the most efficacious, safe, and cost-effective medicines for priority conditions and has the minimum medicines required for a basic healthcare system, while the supplementary list contains essential medicines for priority diseases which requires specialized medical care, specialized training or specialized diagnostic or monitoring facilities [1].

The presence of a medicine on the WHO EMLc is however not an assurance of the pharmaceutical quality of the medicine. It is therefore the responsibility of the relevant national and regional medicines regulatory authorities to ensure that medicines within their jurisdiction are of the right quality and stability through the application of integrated pharmacovigilance and drug surveillance systems [2]. The Standard Treatment Guidelines (STG) published by the Ministry of Health, Ghana, under the auspices of the Ghana National Drugs Programme are to ensure, among others, the availability of quality essential medicines for children and adults in all healthcare settings in the country [3]. The STG of Ghana incorporates some essential children's medicines contained in the WHO EMLc and others which are not on the list.

The healthcare system in Ghana has 5 levels of providers comprising health posts, health centers/clinics, district hospitals, regional hospitals, and tertiary or teaching hospitals. Most health facilities are found in the urban areas while the rural areas have poor health infrastructure. The country has 39.3% of the population below 15 years (2010 population census) with infant mortality and under 5 mortality in 2014 at 41 and 60 (per 1000 live births), respectively. In 2012, 5.2% of Ghana's gross domestic product (GDP) was spent on health. In 2014, the top 5 causes of death in all ages were malaria, anaemia, HIV/AIDS, hypertension, and pneumonia (The Health Sector in Ghana: Facts and Figures, 2015). The Ashanti Region is the most populous region of Ghana, with a projected population of 5,461,534 (GSS 2010 Census). The region has many health facilities including 141 health centers, 116 clinics, 119 hospitals, and 2,241 registered medicine outlets comprising hospital dispensaries, community pharmacies, and licensed chemical shops.

The quality of children's medicines available in medicines outlets in a country directly affects the quality of healthcare available to children in the country. Children are more vulnerable to the adverse effects of poor quality medicines due to their physiological differences from adults and the changes of their medicines disposition during maturation. The necessity of using quality medicines in health delivery systems cannot be overemphasized as poor quality medicines usage, particularly in children, can cause treatment failures, emergence of drug resistance, prolonged sickness, increased healthcare costs, and death [4]. Thus, the use of poor quality medicines in children will result in increased morbidity and mortality.

The quality of medicines can be compromised during manufacture, distribution within country, and storage at elevated temperatures and humidity conditions [5]. There is the need for regular quality assurance and postmarket surveillance of children's medicines by regulatory and allied authorities to ensure the quality of these medicines. However, medicines quality monitoring remains a big challenge in most developing countries due to inadequate facilities and trained personnel and poor regulatory mechanisms [6].

There are very few reported studies on the quality testing of child-specific paediatric formulations [7–10]. Thus, most of the studies on the pharmaceutical quality of children's medicines reported in the literature are essentially subsets of larger medicines quality studies [2]. An evaluation of the physicochemical properties of four paediatric dihydroartemisinin-piperaquine powder for suspension revealed satisfactory drug content with pH: 3.19–4.65, moisture content: 2.6–4.4%, total solids: 86.2–97.2%, and viscosity: 78.5–125.8 mPa·s [11]. An assessment of the quality of artemether-lumefantrine preparations and artemether injections in Cape Coast Metropolis, Ghana, showed that 7 (87.5%) of the 8 brands (6 tablets, 2 suspensions) and all 2 artemether injections passed the International Pharmacopoeia content test [12]. A new validated high performance liquid chromatography method used to analyse the content of 24 artemether-lumefantrine tablets and powders for reconstitution into paediatric suspensions obtained in Ghana showed that all 4 samples which failed (16.7%) were powders for reconstitution into suspensions with two (8.3%) of the samples showing unacceptably higher levels of 222% and 756% artemether [13]. The analysis of microbial load of seven brands of multivitamin syrups marketed in Maiduguri, Nigeria, using the pour-plate method showed that one brand (14.3%) contained the pathogenic bacteria* Salmonella* spp. while the other 6 samples (85.7%) satisfied the microbial load limit of syrups [10].

A study in Kenya showed that 16 out of 23 amodiaquine suspensions (69.6%) sampled from the private retail sector passed the USP limits for content of amodiaquine of 93–107% while 7 samples (30.4%) failed. Also, out of 27 sulphadoxine (SDX)-pyrimethamine (PMT) suspensions analysed for content, 16 samples (59.3%) failed to meet the USP limits for content of SDX and PMT of 90–110% [14]. Eight percent of amoxicillin suspensions purchased from some Arab countries and analysed for drug content using validated chromatographic method after reconstitution failed the pharmacopoeial limits while 38% failed the drug content test after 7 or 14 days after reconstitution [15]. A quality evaluation of 10 metronidazole suspensions in Bangladesh found that 8 of the samples met the BP specification for potency while 2 brands were less potent. Also, all the 10 metronidazole samples satisfied the specification for pH of suspensions [16]. There have also been reports in Nigeria and India of diethylene glycol-induced renal failure and encephalopathy when it was substituted for propylene glycol in paediatric paracetamol formulations [17, 18].

We have previously reported the low average availability of essential children's medicines in the Ashanti Region, Ghana of ~41.3% [19]. The objective of the current study was to evaluate the quality of some essential children's medicines (syrups, suspensions, and solutions) purchased from licensed medicine outlets in the Ashanti Region, Ghana, using the three quality parameters of content of active pharmaceutical ingredient (API), pH, and microbial load. From the study it would be possible to ascertain the proportion of children's medicines that are of good quality and/or substandard and the country of manufacture and level of unlicensed children's medicines available and their possible correlation with product quality.

## 2. Material and Methods

### 2.1. Chemicals and Analytical Reference Standards

Primary analytical reference standards with purity ≥ 99% of ciprofloxacin (CPF), trimethoprim (TMP), sulphamethoxazole (SMZ), metronidazole (MTZ), amoxicillin (AMX), clavulanic acid (CLA), flucloxacillin (FCX), cefuroxime (CFX), arthemether (ART), lumefantrine (LUM), mebendazole (MBZ), albendazole (ALB), ferrous ammonium citrate (FAC), vitamin B1 (VB1), vitamin B3 (VB3), vitamin B6 (VB6), folic acid (FLA), griseofulvin (GFV), paracetamol (PCM), and ibuprofen (IBF) were obtained from the Food and Drugs Authority, Ghana. Analytical grade solvents including sodium hydroxide (BDH, UK), formic acid, methanol, chloroform acetic acid, glacial acetic acid (BDH, UK), ethanol, acetonitrile, ammonium hydroxide reagent ACS, sulphuric acid, ethyl acetate, holmium perchlorate, and potassium dihydrogen orthophosphate (BDH, UK) were used.

### 2.2. Microbiological Media and Test Strains

Buffered sodium chloride-peptone solution pH 7.0 (Sigma-Aldrich, USA), soybean-casein digest agar (Sigma-Aldrich, USA), tryptone soy agar (Fisher Scientific, USA), nutrient agar (BDH, UK), MacConkey agar (BDH, UK), mannitol salt agar (Fisher Scientific, USA), and sterile water were used. Clinical strains of* Escherichia coli, Staphylococcus aureus, Candida albicans, Bacillus subtilis, Pseudomonas aeruginosa, Streptococcus pneumonia*, and* Haemophilus influenza *were used for microbiological analysis.

### 2.3. Sample Collection

Sixty-eight samples of 15 different essential children's medicines employed in the treatment of prevalent diseases in the region and falling into therapeutic groups of antibiotics, anthelmintics, antimalarials, analgesic/anti-inflammatory agents, and antifungals were purchased from hospital dispensaries (24), community pharmacies (24), and licensed chemical sellers shops (35) in the 27 administrative districts of the Ashanti Region, Ghana [19]. Stratified sampling was employed to allocate three paediatric medicines to each district, except the Kumasi Metropolis which, being the best endowed with medicines outlets, was allocated 5 medicines. Random sampling was then used to determine the specific medicine outlets to visit to purchase the medicines in each district for the study from a list of 2,241 registered medicine outlets in the region. The same investigator purchased all the medicines from the selected licensed medicine outlets. For each selected drug, one proprietary product and two or more generics (*n* = 3), depending on availability, were purchased from the medicine outlet for the study. The characteristics of the sampled children's medicines are shown in Table 1. The samples were collected between June 2014 and August 2016 and quality assessment studies were conducted at least six months before their expiry dates.

### 2.4. Determination of Content of API by Ultraviolet Spectroscopy

The percentage content of APIs of cotrimoxazole suspension (trimethoprim TMP and sulfamethoxazole SMZ), metronidazole suspension, cefuroxime suspension, mebendazole suspension, ferrous ammonium citrate solution, paracetamol suspension, and griseofulvin suspension (*n* = 3) was determined by ultraviolet spectrophotometry (UV-VIS spectrophotometer 3000+, Labindia Instruments Pvt Ltd., India) using the standard British Pharmacopoeia methods [20]. The suspensions were shaken to make sure that there were no cakes at the bottom of the bottles. Powders for reconstitution into suspensions were constituted with distilled water as recommended and shaken to ensure content uniformity before samples were taken. The API contents of the paediatric medicines were determined by measuring the absorbance of the appropriately diluted medicines at the following absorbance values: TMP: 204 nm; SMZ: 257 nm; metronidazole: 277 nm; cefuroxime: 274 nm; mebendazole: 305 nm; ferrous ammonium citrate: 472 nm; paracetamol: 257 nm; and griseofulvin: 291 nm.

### 2.5. Determination of Content of API by High Performance Liquid Chromatography

Standard United States Pharmacopeia high performance liquid chromatography (HPLC) methods [21] were used to determine the percentage content of APIs in the suspensions of ciprofloxacin, amoxicillin, flucloxacillin, albendazole, ibuprofen, artemether-lumefantrine, and syrups of multivitamin and folic acid (*n* = 3). The HPLC analyses were undertaken on an Agilent 110 HPLC system (Agilent Technologies, UK) with a stainless steel Agilent Zorbax C18 column (25 cm × 4.6 mm), packed with octadecylsilyl silica gel. The injection volume, flow rate, and wavelength of detection were, respectively, as follows: ciprofloxacin: 10 *μ*l, 1.5 ml/min, 278 nm; amoxicillin: 10 *μ*l, 1.5 ml/min, 230 nm; flucloxacillin: 10 *μ*l, 1.0 ml/min, 225 nm; albendazole: 20 *μ*l, 2.3 ml/min, 308 nm; ibuprofen: 10 *μ*l, 2.3 ml/min, 220 nm; artemether-lumefantrine: 20 *μ*l, 1.0 ml/min, 210 and 380 nm; multivitamin: 20 *μ*l, 1.5 ml/min, 275 nm; and folic acid: 20 *μ*l, 2.0 ml/min, 283 nm. Column temperature was maintained at 25–40°C.

### 2.6. Determination of pH of Drug Samples

The pH of each sample, diluted according to the manufacturer's instruction, was determined with a calibrated Hanna pH meter (model HI 2210, Hanna Instruments, UK). The results were compared with the British Pharmacopoeia [20] pH specifications of each sample.

### 2.7. Microbial Limit Tests

The pour-plate method was used to determine the microbial load of the samples according to British Pharmacopoeia specifications [20].

## 3. Results

Sixty-eight out of 83 samples of children's medicines allocated to the 27 administrative districts of the Ashanti Region, Ghana, were evaluated in the three quality parameters of content of API, pH, and microbial load. Thus, the quality of 68 samples of 15 different essential children's medicines comprising both locally manufactured and imported products were evaluated. The products studied fell into the therapeutic areas of antibiotics (29), antimalarials (4), anthelmintics (13), antifungals (3), haematinics (7), and analgesic/anti-inflammatory agents (12). The imported products were sourced mainly from India and the United Kingdom, showing the importance of these countries as the primary source of imported paediatric medicines in the region. Only one (1.5%) of the samples studied was sourced from another African country, South Africa. This observation could be attributed to the low production capacity for children's medicines by the nascent pharmaceutical manufacturing industries in Africa.

Thirty-six (52.9%) of the products possessed the Food and Drugs Authority, Ghana, or foreign product manufacturing license (PML) numbers while the remaining 32 (47.1%) had no product registration numbers. Thus, a large proportion of the essential children's medicines sampled had not been properly licensed by the national medicines regulatory authority for use in Ghana. Some prescription-only children's medicines were available in some licensed chemical sellers shops even though they are licensed to supply only over-the-counter medicines. All the products had the product strength, country of manufacture, manufacturing date, and expiry date on the product labels (Table 1). The shelf lives of the products ranged between 1 and 5 years. All the products had batch numbers except CPF2 (1.5%).

The results of the quality assessment of the sampled children's medicines in terms of percentage content of active pharmaceutical ingredient(s), pH, and microbial load and content are shown in Table 2. The percentage drug contents were determined using either high performance liquid chromatography or ultraviolet spectroscopy methods and were calculated relative to the labelled amount of the drug. The retention times of samples assayed by HPLC varied with the type of drug analysed and were similar for different brands of the same children's medicine. This observation confirms the authenticity of the API(s) contained in the medicines. The two samples of ciprofloxacin oral suspensions (retention time 8.73 min) had drug contents far below the minimum of the official specification of 98–102% and are therefore substandard. One ciprofloxacin sample passed the pH test while the other failed. However, both ciprofloxacin samples passed the microbial limit test and could pose no microbial health hazard.

All the cotrimoxazole suspensions sampled failed the official drug content test but were compliant with specifications for pH and microbial load and content. Cotrimoxazole suspensions contain the APIs trimethoprim (TMP) and sulphamethoxazole (SMZ) with specifications of 92.5–107.5% for the two drugs. Cotrimoxazole suspensions CTX1, CTX2, and CTX4 contained the right amounts of SMZ but had amounts of TMP higher than the specified upper limits. Samples CTX3 and CTX5, however, had both TMP and SMZ above the upper limits. Thus all the cotrimoxazole suspensions sampled are substandard products. Metronidazole oral suspensions are specified to contain 95–105% of the drug. Only sample MTZ4 (16.7%) passed the test while MTZ7 failed marginally. All the six samples, however, passed the pH and microbial limit tests. Samples MTZ2, MTZ3 and MTZ5 contained much lower amounts of the drug than stipulated and their use could result in serious underdosage and possible therapeutic failure.

The amoxicillin suspensions analysed by HPLC had retention times of 3.65–4.04 min. All the six amoxicillin suspension samples, including four locally manufactured products, satisfied the compendial requirements for drug content, pH, and microbial quality and are thus deemed to be good quality. The flucloxacillin suspension samples were analysed by HPLC and had retention time of 2.36 min. All the flucloxacillin samples failed the content of API test but passed both the pH and the microbial limit tests. The samples had their contents of API far below the specified lower limit and are substandard. Only one cefuroxime suspension sample passed the content of API test while the remaining samples failed the test. Cefuroxime suspension CFX1 failed the content of API, pH, and microbial limit tests while samples CHX2-CFX6 passed both the pH and the microbial limit tests. Sample CFX1 contained the pathogenic bacteria* Staphylococcus aureus* which could adversely affect the health of children. This observation clearly shows that the cefuroxime suspensions available in the region are of poor quality and unsuitable for use.

The retention times of artemether and lumefantrine analysed by HPLC ranged from 3.23 to 4.07 min and from 3.91 to 14.21 min, respectively. None of the artemether-lumefantrine suspensions satisfied the official specifications for content of artemether and lumefantrine of 90–110%. Sample ATL1 passed the drug content test for lumefantrine but failed the test for artemether. The other artemether-lumefantrine suspensions failed the content tests for both artemether and lumefantrine. All the samples passed the microbial limit test but only sample ATL1 passed the pH test while the rest were noncompliant. Only one mebendazole sample (MBZ3) complied with specifications for drug content, pH, and microbial load and is of good quality for use. Sample MBZ5 failed the microbial limit test with the total aerobic microbial count far exceeding the official limit of 1000 cfu/ml. Also, while sample MBZ2 failed the pH test the remaining samples passed the test.

The retention times of the albendazole suspensions analysed by HPLC ranged from 7.23 to 7.28 minutes. Six of the samples (75.0%) passed the content of API test while the rest which failed had drug contents above the upper limit of the official range of 90–110%. All the albendazole suspension samples complied with the microbial load test but only half the number of samples passed the pH test. Only albendazole samples ABZ3 and ABZ4 complied with official specifications for content of API, pH, and microbial load. Two of the three griseofulvin oral suspensions failed the content of API test, with one of them GSV3 failing marginally. All the griseofulvin samples passed the microbial limit test but two of the samples failed the pH test. All the three ferrous sulphate syrups analysed complied with the content of API and microbial limit tests but one sample failed the pH test.

The two multivitamin syrups analysed by HPLC had the following retention times: Vitamin B1 (2.06–2.75 min), Vitamin B2 (6.84 min), Vitamin B3 (2.74–3.17 min), and Vitamin B6 (1.97 min). Multivitamin syrup MVM1 was noncompliant for the requirements of content of vitamins B1, B2, B3, and B6. Multivitamin syrup MVM2 complied with specifications for content of vitamin B1 but was noncompliant for content of vitamins B3 and B6. Samples MVM1 and MVM2 contained unacceptably low amounts of vitamin B1 and vitamin B6, respectively. However, the two multivitamin syrup samples passed both the pH and the microbial limit tests. The two folic acid syrups analysed (retention time: 3.83 min) failed both the content of API and the pH tests but passed the microbial limit test. Three (42.9%) paracetamol oral suspension samples analysed passed the content of API test, with three of the samples failing marginally. All the seven paracetamol samples passed the microbial load test while six (85.7%) passed the pH test. Four ibuprofen oral suspensions analysed by HPLC (retention times: 19.56–20.43 min) complied with the official specification for content of API while all the samples passed the microbial limit test. However, three of the samples failed the pH test while two passed.

Table 3 presents a summary of the results of the drug content determination, pH, and microbial limit tests for the children's medicines studied. In all, 26 (38.2%) of the samples studied complied with official specifications for content of API(s) while 42 (61.8%) were noncompliant. Also, 49 (72.1%) of the samples passed the pH test while 19 (27.9%) failed the test. Two (2.9%) of the children's medicines sampled were noncompliant with specifications for microbial load and content while 66 (97.1%) were compliant. In all, eighteen (26.5%) samples passed all the three quality evaluation tests while one sample (CFX1) failed all the tests.  Table 4 shows a comparative analysis of the quality of locally manufactured children's medicines versus foreign-produced samples. Thirteen (36.1%) of the locally manufactured children's medicine samples passed the content of API test while twenty-three (63.9) failed. Also, thirteen (40.6%) of the foreign-manufactured products passed the content of API test while nineteen (59.4%) failed. Thus the failure rate of the foreign-manufactured products was similar to the locally manufactured products. In terms of compliance with pH, the LMP performed marginally better than FMP. All the FMP sampled were compliant with the microbial load and content test while two (5.6%) of the LMP failed.  Table 5 shows the effect of registration status/licensing on the quality parameters assessed. Whereas the registered products had higher pass rates for pH and microbial load, the unregistered products showed higher pass rates for content of API. Also, the menace of poor quality children's medicines was encountered irrespective of the country of manufacture of the product (Table 6).

## 4. Discussion

Children require safe, effective, and quality paediatric pharmaceutical formulations for the management and treatment of diseases to cater for the differences between children and adults regarding drug metabolism and treatment responses [22]. Furthermore, the use of converted adult dosages of medicines for children is discouraged due to the potential for over- and underdosages leading to harmful adverse clinical effects.

The quality of the children's medicines was assessed using the three quality parameters of content of active pharmaceutical ingredient (API), pH, and microbial load. Inadequate amounts of API will result in underdosed medication, leading to poor treatment outcomes while excessive amounts of API cause overdosage of medication, leading to increased adverse drug reactions and treatment failure. The pH of liquid children's medicines is important in assuring the quality, stability, and efficacy of medicines. Medicines with high microbial load due to product contamination and poor quality assurance during manufacturing pose formidable health risks, particularly to children. The presence of pathogenic bacteria such as* Escherichia coli, Staphylococcus aureus* and* Pseudomonas aeruginosa* can cause serious microbial infections such as pneumonia and eye infections in children. While all the three quality parameters assessed in the current study are important to the overall quality of children's medicines, the content of API is the most significant quality parameter which ultimately determines the attainment of positive treatment outcomes or treatment failure.

In the current study, less than one-third of the products sampled passed all the three quality evaluation tests and were considered to be of good quality. The rest of the children's medicines were deemed to be poor quality medicines. Poor quality medicines may be classified into substandard (manufacturing errors), falsified (including counterfeit), or degraded (loss of activity due to poor storage of product in supply chain). Most of the paediatric formulations sampled were substandard and therefore of poor quality. Significantly, none of the products sampled was found to be counterfeit or fake. Substandard medicines are genuine medicines which fail to meet either their quality standards or their specifications, or both. On the other hand, counterfeit medicines are medicines deliberately and fraudulently mislabeled with respect to identity and/or source and may contain the correct ingredients, wrong ingredients, no ingredients, or insufficient ingredients or have fake packaging [23].

The prevalence of poor quality children's medicines was comparable between locally manufactured and foreign-produced (imported) medicines. This observation runs counter to the general perception that imported medicines are of better quality than those manufactured locally in developing and other resource-poor countries. There also appeared to be no correlation between the registration status/licensing of children's medicines and product quality.

The use of poor quality paediatric medicines can lead to increased child mortality and morbidity, increased adverse reactions, and the development of drug resistance [4]. Even though the reasons for the high substandard nature of the medicines were not determined, the high failure rates could be attributed to poor manufacturing and possibly poor storage of the essential medicines in the supply chain. The rate of decomposition of the API in medicines is accelerated in high temperature and humidity conditions [6]. There is therefore the need for manufacturers of these paediatric pharmaceutical formulations to adhere strictly to current good manufacturing practices (cGMP). Also, manufacturers, wholesalers, and retailers of children's medicines should adhere to the recommended storage conditions to reduce the incidence of physical and chemical degradation of the products.

The microbial quality of the paediatric formulations studied was generally good. There is however the need for continuous evaluation of the microbial quality of paediatric syrups and suspensions since microbial and fungal growth in such dosage forms increases with time and can threaten the life of immunocompromised children [24]. The stability of pH of a liquid formulation over time provides an indication of the microbial quality and chemical stability of the product. Poor storage conditions and the formulation of unstable products can have adverse effects on pH as well as on the physical and chemical stability of children's medicines.

In Ghana, the Food and Drugs Authority (FDA), formerly the Food and Drugs Board, was established in 1997 under the Food and Drugs Law, 1992 (PNDCL 305B). The FDA is the medicines regulatory authority (MRA) of Ghana with the objective of providing and enforcing standards for the sale of food, herbal medicinal products, cosmetics, drugs, medical devices, and household chemical substances. The presence of so many unregistered children's medicines in the study area denotes inadequate regulatory and surveillance activities of the FDA, Ghana. The absence of PML numbers on some products could be due to neglect associated with poor product labelling by the manufacturers involved as none of such products was found to be counterfeit. To mitigate the menace of unlicensed/unregistered medicines in Ghana, the many entry points of the country's borders should be properly manned with adequate trained FDA, Ghana personnel to stem the infiltration of unregistered and counterfeit medicines from neighboring countries into Ghana.

The menace of poor quality children's medicines in Ghana and other developing and resource-poor countries can be reduced through the strengthening of the national medicines regulatory authorities (MRAs) and enhancement in the quality of medicines manufacturing. The MRAs could be strengthened through the provision of adequate financial and logistical resources; enforcement of existing legislation; provision of medicines analytical equipment/laboratories; recruitment of adequate qualified staff; and enhancement of conditions of service to attract and retain qualified staff. Also, the quality of medicines produced by medicines manufacturers in resource-poor countries could be enhanced through the recruitment and retention of qualified manufacturing staff, for example, pharmaceutical technologists, analysts, quality control personnel; adherence to quality assurance protocols, and adequate regulatory control.

In Ghana, licensed chemical shops, unlike pharmacies, are medicine outlets which are authorized to engage in the retail supply of only over-the-counter medicines or class C medicines. The availability of some prescription-only children's medicines in some licensed chemical shops is therefore a gross abuse of the relevant regulations by proprietors of such outlets and an indication of regulatory failure by the Pharmacy Council, Ghana, the statutory regulatory body established by the Pharmacy Act, 1994 (Act 489). The major function of the Pharmacy Council is to “secure in the public interest the highest standards in the practice of pharmacy” in Ghana and its mission is to “secure the highest level of pharmaceutical care by ensuring competent pharmaceutical care providers who practice within agreed standards and are accessible to the whole population.” The Pharmacy Council should enhance its monitoring activities of all medicine outlets in the country to ensure compliance with existing rules, regulations, and standards. It is also pertinent for the Food and Drugs Authority and the Pharmacy Council to collaborate and work together to enhance their medicines surveillance and regulatory activities to ensure that only licensed, good quality medicines are made available to the Ghanaian people and that pharmaceutical care providers are in compliance with existing standards and rules and regulations.

## 5. Conclusions

The results from the study clearly show that a significant proportion of the children's medicines available in the Ashanti Region, Ghana, are of poor quality. This has serious consequences on the health and general well-being of children in the region. Even though most of the medicines sampled passed the pH and microbial load tests, they also failed the content of API test and were thus of poor quality. Both locally manufactured and imported children's medicines studied demonstrated similar defective quality properties. Also, medicines sourced from the United Kingdom and India were similarly found to be of poor quality. There was no correlation between unlicensed children's medicines and product quality. The administration of poor quality medicines to children will cause treatment failures and increase drug resistance, leading to increased morbidity and mortality. The poor quality of the children's medicines could be attributed generally to inadequate quality assurance processes employed during manufacturing of the products. It is therefore recommended that manufacturers of children's medicines adhere strictly to good manufacturing practices to ensure the production of quality medicines.

## Figures and Tables

**Table 1:** Product characteristics of the essential children's medicines investigated.

Product and code	Labeled strength	Country of manufacture	Batch number	Manufacturing date	Expiry date	FDA/PML number
Ciprofloxacin						
CPF1	250 mg/5 ml	UK	0710069	06/2015	05/2017	Yes
CPF2	250 mg/5 ml	India	-	06/2014	05/2017	No

Cotrimoxazole						
CTX1	125 mg/5 ml	UK	EXMT 002	09/2014	08/2017	No
CTX2	125 mg/5 ml	India	XT 029	02/2015	01/2018	Yes
CTX3	125 mg/5 ml	Ghana	0302R	02/2015	02/2018	No
CTX4	125 mg/5 ml	Ghana	15012	08/2015	08/2018	Yes
CTX5	125 mg/5 ml	Ghana	0250495	05/2015	04/2017	Yes

Metronidazole						
MTZ1	200 mgt/5 ml	South Africa	46159A	12/2014	11/2016	Yes
MTZ2	200 mg/5 ml	Ghana	X-214	03/2015	03/2017	Yes
MTZ3	200 mg/5 ml	UK	MTXT-002	10/2014	09/2017	No
MTZ4	200 mg/5 ml	Ghana	0403R	03/2015	03/2017	No
MTZ5	200 mg/5 ml	Ghana	15002	04/2015	04/2018	Yes
MTZ6	200 mg/5 ml	Ghana	0260385	07/2015	06/2017	Yes

Amoxicillin						
AMX1	125 mg/5 ml	UK	676164	11/2013	11/2018	No
AMX2	125 mg/5 ml	UK	13305001	08/2013	07/2016	No
AMX3	125 mg/5 ml	Ghana	AS159	06/2015	04/2018	Yes
AMX4	125 mg/5 ml	Ghana	0270435	05/2015	04/2017	Yes
AMX5	125 mg/5 ml	Ghana	15009	05/2015	05/2018	Yes
AMX6	125 mg/5 ml	Ghana	2305P	05/2014	05/2016	No

Flucloxacillin						
FCX1	125 mg/5 ml	India	150079	01/2015	12/2017	No
FCX2	125 mg/5 ml	Ghana	2307R	07/2015	07/2017	Yes
FCX3	125 mg/5 ml	Ghana	0390315	09/2015	08/2017	Yes
FCX4	125 mg/5 ml	Ghana	15021	08/2015	08/2018	No

Cefuroxime						
CFX1	125 mg/5 ml	Ghana	4808R	08/2015	08/2017	No
CFX2	125 mg/5 ml	India	BS-59001	07/2015	06/2017	Yes
CFX3	125 mg/5 ml	UK	C703319	10/2014	10/2016	No
CFX4	125 mg/5 ml	India	83	08/2015	07/2017	No
CFX5	125 mg/5 ml	UK	STR44A00	08/2014	08/2016	Yes
CFX6	125 mg/5 m	India	CFL-073	10/2015	10/2017	Yes

Artemether-lumefantrine						
ATL1	20 mg/120 mg/5 ml	India	0633	02/2014	02/2016	Yes
ATL2	20 mg/120 mg/5 ml	Ghana	LG-414	02/2015	01/2017	Yes
ATL3	20 mg/120 mg/5 ml	Ghana	2804R	03/2016	03/2018	No
ATL4	20 mg/120 mg/5 ml	India	3A09150	01/2015	12/2017	Yes

Mebendazole						
MBZ1	100 mg/5 ml	Belgium	DLB 3600	01/2014	12/2018	No
MBZ2	100 mg/5 ml	Ghana	15001	05/2015	05/2018	No
MBZ3	100 mg/5 ml	Ghana	0780165	07/2015	06/2017	No
MBZ4	100 mg/5 ml	Ghana	12	03/2015	03/2018	Yes
MBZ5	100 mg/5 ml	Ghana	M2002	09/2014	08/2017	No

Albendazole						
ABZ1	100 mg/5 ml	India	340960	08/2014	07/2017	No
ABZ2	200 mg/5 ml	India	G4016	06/2014	05/2017	Yes
ABZ3	100 mg/5 ml	India	W150828	08/2015	07/2018	Yes
ABZ4	100 mg/5 ml	India	14328	05/2014	04/2017	Yes
ABZ5	100 mg/5 ml	India	340953	08/2014	07/2017	Yes
ABZ6	100 mg/5 ml	India	T140023	03/2014	02/2017	No
ABZ7	100 mg/5 ml	India	L-564001	02/2014	01/2017	Yes
ABZ8	200 mg/5 ml	India	331134	11/2013	10/2016	No

Griseofulvin						
GFV1	125 mg/5 ml	India	GS 1401	01/2014	02/2016	Yes
GFV2	125 mg/5 ml	India	5E 01045	02/2015	01/2018	Yes
GFV3	125 mg/5 ml	Ghana	001	05/2015	05/2018	No

Ferrous sulphate						
FES1	10 mg/5 ml	Ghana	VL 785	10/2015	09/2017	Yes
FES2	75 mg/5 ml	India	2F140KE	12/2014	11/2016	No
FES3	200 mg/5 ml	India	T140011	07/2014	07/2016	No

Multivitamin						
MVM1	2 mg/5 ml (Vit B1, B2), 2 mcg/5 ml (Vit B3), 0.225 mg/5 ml (Vit B6)	Ghana	AY 231	06/2015	05/2017	Yes
MVM2	2 mg/5 ml (Vit B1), 1.2 mg/5 ml (Vit B3), 0.2 mg/5 ml (Vit B6)	Ghana	0290158	02/2016	02/2017	Yes

Folic acid						
FLA1	500 mcg/5 ml	Ghana	0430025	09/2015	11/2016	No
FLA2	500 mcg/5 ml	Ghana	FO-035	11/2014	10/2016	Yes

Paracetamol						
PCM1	125 mg/5 ml	UK	AS 17120	02/2015	02/2018	No
PCM2	125 mg/5 ml	Ghana	RNA2P018	08/2015	08/2018	Yes
PCM3	125 mg/5 ml	Ghana	3609R	09/2015	09/2018	No
PCM4	125 mg/5 ml	Ghana	10L319	10/2013	09/2016	Yes
PCM5	125 mg/5 ml	Ghana	E 005	10/2015	09/2018	No
PCM6	125 mg/5 ml	Ghana	0361865	10/2015	09/2017	Yes
PCM7	125 mg/5 ml	Ghana	1008F	08/2015	08/2018	Yes

Ibuprofen						
IBF1	100 mg/5 ml	UK	IBXT-002	09/2014	08/2017	No
IBF2	100 mg/5 ml	Ghana	1506P	06/2014	06/2017	No
IBF3	100 mg/5 ml	Ghana	E001	04/2015	04/2017	No
IBF4	100 mg/5 ml	India	IB4 001	09/2014	08/2017	No
IBF5	100 mg/5 ml	Ghana	1030025	09/2014	08/2017	Yes

(a) CPF = ciprofloxacin suspension, CTX = cotrimoxazole suspension, MTZ = metronidazole suspension, AMX = amoxicillin suspension, FCX = flucloxacillin suspension, CFX = cefuroxime suspension, ATL = Artemether-lumefantrine suspension, MBZ = mebendazole suspension, ABZ = albendazole suspension, GFV = griseofulvin suspension, FES = ferrous sulphate syrup, MVM = multivitamin syrup, FLA = folic acid suspension, PCM = paracetamol syrup, and IBF = ibuprofen suspension; (b) FDA = Food and Drugs Authority, Ghana; PML =p manufacturing license; (c) the numbers indicated against a particular product are different brands of the same drug.

**Table 2:** Results of content of API, pH, and microbial load of the essential children's medicines investigated.

Product and Code	Retention time (min)	^*∗*^Content of API (%)	Remarks on content of API (%)	^*∗∗*^pH	Remarks on pH	Microbial load
^+^Ciprofloxacin						
CFX1	8.73	72.14	Failed	10.24	Failed	Passed
CFX2	8.73	67.45	Failed	4.52	Passed	Passed

Cotrimoxazole						
CTX1	n/a	110.94/102.69	Failed	5.46	Passed	Passed
CTX2	n/a	112.50/100.00	Failed	5.30	Passed	Passed
CTX3	n/a	134.00/123.00	Failed	5.16	Passed	Passed
CTX4	n/a	122.00/93.75	Failed	5.66	Passed	Passed
CTX5	n/a	118.13/124.75	Failed	5.57	Passed	Passed
		(TMP/SMZ)				

Metronidazole						
MTZ1	n/a	113.60	Failed	6.56	Passed	Passed
MTZ2	n/a	91.50	Failed	5.42	Passed	Passed
MTZ3	n/a	90.00	Failed	4.95	Passed	Passed
MTZ4	n/a	95.60	Passed	6.28	Passed	Passed
MTZ5	n/a	69.70	Failed	5.15	Passed	Passed
MTZ6	n/a	106.70	Failed	6.06	Passed	Passed

^+^Amoxicillin						
AMX1	4.04	85.74	Passed	4.84	Passed	Passed
AMX2	3.65	95.46	Passed	5.14	Passed	Passed
AMX3	3.68	95.70	Passed	5.57	Passed	Passed
AMX4	4.04	88.12	Passed	4.75	Passed	Passed
AMX5	4.05	103.58	Passed	5.73	Passed	Passed
AMX6	3.67	94.07	Passed	4.65	Passed	Passed

^+^Flucloxacillin						
FCX1	2.37	53.84	Failed	4.75	Passed	Passed
FCX2	2.36	35.10	Failed	4.47	Passed	Passed
FCX3	2.36	60.01	Failed	4.78	Passed	Passed
FCX4	2.36	46.12	Failed	5.13	Passed	Passed

Cefuroxime						
CFX1	n/a	45.43	Failed	3.18	Failed	^∧^Failed
CFX2	n/a	74.90	Failed	4.1	Passed	Passed
CFX3	n/a	58.60	Failed	4.06	Passed	Passed
CFX4	n/a	96.70	Passed	3.7	Passed	Passed
CFX5	n/a	135.00	Failed	4.75	Passed	Passed
CFX6	n/a	90.10	Failed	4.98	Passed	Passed

^+^Artemether-lumefantrine						
ATL1	4.07/5.80	141.99/97.13	Failed	4.88	Passed	Passed
ATL2	3.30/14.21	84.82/125.85	Failed	3.84	Failed	Passed
ATL3	3.23/3.91	153.43/45.32	Failed	3.70	Failed	Passed
ATL4	3.35/14.18	209.67/127.68	Failed	2.88	Failed	Passed

Mebendazole						
MBZ1	n/a	111.97	Failed	5.02	Passed	Passed
MBZ2	n/a	85.91	Failed	7.30	Failed	Passed
MBZ3	n/a	93.60	Passed	5.07	Passed	Passed
MBZ4	n/a	72.80	Failed	5.37	Passed	Passed
MBZ5	n/a	89.00	Failed	5.46	Passed	^∧∧^Failed

^+^Albendazole						
ABZ1	7.25	106.80	Passed	4.42	Failed	Passed
ABZ2	7.26	102.60	Passed	4.08	Failed	Passed
ABZ3	7.25	93.11	Passed	4.52	Passed	Passed
ABZ4	7.25	95.59	Passed	4.50	Passed	Passed
ABZ5	7.23	97.64	Passed	4.38	Failed	Passed
ABZ6	7.23	103.37	Passed	4.29	Failed	Passed
ABZ7	7.24	130.54	Failed	4.61	Passed	Passed
ABZ8	7.28	188.45	Failed	4.60	Passed	Passed

Griseofulvin						
GSV1	n/a	105.10	Passed	4.96	Failed	Passed
GSV2	n/a	70.85	Failed	4.96	Failed	Passed
GSV3	n/a	94.31	Failed	5.67	Passed	Passed

Ferrous sulphate						
FES1	n/a	97.50	Passed	6.07	Passed	Passed
FES2	n/a	104.60	Passed	3.81	Failed	Passed
FES3	n/a	106.70	Passed	5.44	Passed	Passed

^+^Multivitamin						
MVM1				2.50	Passed	Passed
(i) Vit B1	2.06	120.00	Failed			
(ii) Vit B2	6.84	25.33	Failed			
(iii) Vit B3	2.74	113.68	Failed			
(iv) Vit B6	1.97	88.16	Failed			
MVM2				2.69	Passed	Passed
(i) Vit B1	2.75	99.41	Passed			
(ii) Vit B2	-	-	-			
(iii) Vit B3	3.17	129.7	Failed			
(iv) Vit B6	1.97	12.58	Failed			

^+^Folic acid						
FLA1	3.83	71.40	Failed	3.59	Failed	Passed
FLA2	3.83	68.38	Failed	4.93	Failed	Passed

Paracetamol						
PCM1	n/a	89.00	Failed	4.92	Passed	Passed
PCM2	n/a	97.40	Passed	4.17	Passed	Passed
PCM3	n/a	108.00	Failed	4.10	Passed	Passed
PCM4	n/a	106.80	Failed	4.34	Passed	Passed
PCM5	n/a	101.70	Passed	4.71	Passed	Passed
PCM6	n/a	102.80	Passed	5.18	Passed	Passed
PCM7	n/a	105.40	Failed	7.20	Failed	Passed

^+^Ibuprofen						
IBF1	19.73	98.48	Passed	4.27	Passed	Passed
IBF2	19.56	100.97	Passed	4.78	Failed	Passed
IBF3	20.34	99.28	Passed	5.88	Failed	Passed
IBF4	19.59	119.87	Failed	5.62	Failed	Passed
IBF5	20.43	99.24	Passed	4.40	Passed	Passed

(a) + denotes samples analysed by high performance liquid chromatography, otherwise samples were analysed by ultraviolet spectrophotometry; (b) TMP = trimethoprim; SMZ = sulphamethoxazole; (c) ^*∗*^Acceptance criteria: ciprofloxacin suspension (98–102%), co-trimoxazole suspension (TMP & SMZ: 92.5–107.5%), metronidazole suspension (95–105%), amoxicillin suspension (80–120%), flucloxacillin suspension (80–120%), cefuroxime suspension (95–105%), artemether-lumefantrine (90–110%), mebendazole suspension (90–110%), albendazole suspension (90–110%), griseofulvin suspension (95–105%), ferrous sulphate syrup (95–110%), Vitamins B1, B2, B3 and B6 (90–106%, 90–110%, 95–105% and 90–110%, respectively), folic acid syrup (90–110%), paracetamol syrup (95–105%), ibuprofen syrup (95–105%); (d) ^*∗∗*^Acceptance criteria: ciprofloxacin suspension (3.0–4.5), co-trimoxazole suspension (5.0–6.5), metronidazole suspension (5.0–6.5), amoxicillin suspension (4.0–7.0), flucloxacillin suspension (4.0–7.0), cefuroxime suspension (3.5–7.0), artemether-lumefantrine (4.5–6.8), mebendazole suspension (4.5–5.5), albendazole suspension (4.5–5.5), griseofulvin suspension (5.5–7.5),: ferrous sulphate syrup (5.2–6.5), multivitamin syrup (2.5–4.0), folic acid suspension (5.2–6.50), paracetamol syrup (4.0–6.9), ibuprofen suspension (3.6–4.6); (e) ^∧^Staphylococcus* aureus* was isolated; (f) ^∧∧^Total aerobic microbial count of 1012 cfu/ml, exceeding the BP (2013) limit of ≤1000 cfu/ml.

**Table 3:** Summary results of the quality of essential children's medicines investigated based on content of API, pH, and microbial load.

Type of essential children's medicine	Number of samples analysed	Content of API		pH		Microbial load	
		Pass	Fail	Pass	Fail	Pass	Fail
Ciprofloxacin	2	0 (0.0)	2 (100.0)	1 (50.0)	1 (50.0)	2 (100.0)	0 (0.0)
Cotrimoxazole	5	0 (0.0)	5 (100.0)	5 (100.0)	0 (0.0)	5 (100.0)	0 (0.0)
Metronidazole	6	1 (16.7)	5 (83.3)	6 (100.0)	0 (0.0)	6 (100.0)	0 (0.0)
Amoxicillin	6	6 (100.0)	0 (0.0)	6 (100.0)	0 (0.0)	6 (100.0)	0 (0.0)
Flucloxacillin	4	0 (0.0)	4 (100.0)	4 (100.0)	0 (0.0)	4 (100.0)	0 (0.0)
Cefuroxime	6	1 (16.7)	5 (83.3)	5 (83.3)	1 (16.7)	5 (83.3)	1 (16.7)
Artemether-lumefantrine	4	0 (0.0)	4 (100.0)	1 (25.0)	3 (75.0)	4 (100.0)	0 (0.0)
Mebendazole	5	1 (20.0)	4 (80.0)	4 (80.0)	1 (20.0)	4 (80.0)	1 (20.0)
Albendazole	8	6 (75.0)	2 (25.0)	4 (50.0)	4 (50.0)	8 (100.0)	0 (0.0)
Griseofulvin	3	1 (33.3)	2 (66.7)	1 (33.3)	2 (66.7)	3 (100.0)	0 (0.0)
Ferrous sulphate	3	3 (100.0)	0 (0.0)	2 (66.7)	1 (33.3)	3 (100.0)	0 (0.0)
Multivitamin	2	0 (0.0)	2 (100.0)	2 (100.0)	0 (0.0)	2 (100.0)	0 (0.0)
Folic acid	2	0 (0.0)	2 (100.0)	0 (0.0)	2 (100.0)	2 (100.0)	0 (0.0)
Paracetamol	7	3 (42.9)	4 (57.1)	6 (85.7)	1 (14.3)	7 (100.0)	0 (0.0)
Ibuprofen	5	4 (80.0)	1 (20.0)	2 (40.0)	3 (60.0)	5 (100.0)	0 (0.0)

Total	68	26 (38.2)	42 (61.8)	49 (72.1)	19 (27.9)	66 (97.1)	2 (2.9)

Values for pass and fail provide the number of samples in each category and the number in parenthesis is the percentage of pass or fail.

**Table 4:** Summary results of the quality of locally manufactured and foreign (imported) essential children's medicines investigated.

Source of essential children's medicines	Number of samples analyzed	Content of API (%)		pH		Microbial load	
		Pass	Fail	Pass	Fail	Pass	Fail
Locally manufactured	36	13 (36.1)	23 (63.9)	27 (75.0)	9 (25.0)	34 (94.4)	2 (5.6)
Foreign-produced	32	13 (40.6)	19 (59.4)	22 (68.8)	10 (31.2)	32 (100.0)	0 (0.0)

Values for pass and fail provide the number of samples in each category and the number in parenthesis is the percentage pass or fail.

**Table 5:** Summary results of the quality of sampled essential children's medicines with and without Food and Drugs Authority, Ghana, and/or Product Manufacturing License (PML) numbers.

Registration status of essential children's medicines	Number of samples analyzed	Content of API (%)		pH		Microbial load	
		Pass	Fail	Pass	Fail	Pass	Fail
FDA/PML number	36	12 (33.3)	24 (66.7)	27 (75.0)	9 (25.0)	36 (100.0)	0 (0.0)
No registration number	32	14 (43.7)	18 (56.3)	22 (68.8)	10 (31.2)	30 (93.7)	0 (6.3)

**Table 6:** Summary results of the quality of sampled essential children's medicines based on country of manufacture.

Country of manufacture of essential children's medicines	Number of samples analyzed	Content of API (%)		pH		Microbial load	
		Pass	Fail	Pass	Fail	Pass	Fail
Ghana	36	13 (36.1)	23 (63.9)	27 (75.0)	9 (25.0)	34 (94.4)	2 (5.6)
India	21	10 (47.6)	11 (52.4)	12 (57.1)	9 (42.9)	21 (100.0)	0 (0.0)
United Kingdom	9	3 (33.3)	6 (66.7)	8 (88.9)	1 (11.1)	9 (100.0)	0 (0.0)
Belgium	1	0 (0.0)	1 (100.0)	1 (100.0)	0 (0.0)	1 (100.0)	0 (0.0)
South Africa	1	0 (0.0)	1 (100.0)	1 (100.0)	0 (0.0)	1 (100.0)	0 (0.0)
